# Shewanella oneidensis MR-1 Utilizes both Sodium- and Proton-Pumping NADH Dehydrogenases during Aerobic Growth

**DOI:** 10.1128/AEM.00415-18

**Published:** 2018-05-31

**Authors:** Kody L. Duhl, Nicholas M. Tefft, Michaela A. TerAvest

**Affiliations:** a Department of Biochemistry and Molecular Biology, Michigan State University, East Lansing, Michigan, USA; University of Bayreuth

**Keywords:** NADH dehydrogenase, Shewanella, bioenergetics, respiration

## Abstract

Shewanella oneidensis MR-1 is a metal-reducing bacterium with the ability to utilize many different terminal electron acceptors, including oxygen and solid-metal oxides. Both metal oxide reduction and aerobic respiration have been studied extensively in this organism. However, electron transport chain processes upstream of the terminal oxidoreductases have been relatively understudied in this organism, especially electron transfer from NADH to respiratory quinones. Genome annotation indicates that S. oneidensis MR-1 encodes four NADH dehydrogenases, a proton-translocating dehydrogenase (Nuo), two sodium ion-translocating dehydrogenases (Nqr1 and Nqr2), and an “uncoupling” dehydrogenase (Ndh), but none of these complexes have been studied. Therefore, we conducted a study specifically focused on the effects of individual NADH dehydrogenase knockouts in S. oneidensis MR-1. We observed that two of the single-mutant strains, the Δ*nuoN* and Δ*nqrF1* mutants, exhibited significant growth defects compared with the wild type. However, the defects were minor and only apparent under certain growth conditions. Further testing of the Δ*nuoN* Δ*nqrF1* double-mutant strain yielded no growth in minimal medium under oxic conditions, indicating that Nuo and Nqr1 have overlapping functions, but at least one is necessary for aerobic growth. Coutilization of proton- and sodium ion-dependent energetics has important implications for the growth of this organism in environments with varied pH and salinity, including microbial electrochemical systems.

**IMPORTANCE** Bacteria utilize a wide variety of metabolic pathways that allow them to take advantage of different energy sources, and to do so with varied efficiency. The efficiency of a metabolic process determines the growth yield of an organism, or the amount of biomass it produces per amount of substrate consumed. This parameter has important implications in biotechnology and wastewater treatment, where low growth yields are often preferred to minimize the production of microbial biomass. In this study, we investigated respiratory pathways containing NADH dehydrogenases with varied efficiency (i.e., the number of ions translocated per NADH oxidized) in the metal-reducing bacterium Shewanella oneidensis MR-1. We observed that two different respiratory pathways are used concurrently, and at least one pathway must be functional for growth under oxic conditions.

## INTRODUCTION

Shewanella oneidensis MR-1 is a facultative anaerobe with the capability to respire using a wide variety of terminal electron acceptors in the absence of oxygen (1). One of the best-studied aspects of this organism is its use of solid-metal oxides and electrodes as terminal electron acceptors (2). S. oneidensis MR-1 interacts with solid electron acceptors via the Mtr pathway, which transfers electrons to the acceptor either through direct contact or soluble flavin electron shuttles (3–5). This capability is useful for many different bioelectrochemical technologies (6–9). For example, S. oneidensis MR-1 has been engineered to act as a biosensor by linking Mtr expression, and therefore electric current generation, to a chemical signal in the environment (10, 11). “Unbalanced fermentation” has also been developed in S. oneidensis MR-1, allowing it to overcome redox imbalance between substrates and products by releasing excess reducing equivalents to an anode electrode via the Mtr pathway (12).

While the Mtr pathway and other terminal oxidoreductases are well studied, upstream processes that transfer electrons into the respiratory quinol pool are less well understood. S. oneidensis MR-1 uses a variety of complexes to transfer electrons to the quinol pool, and these may use one of several different electron donors, including primary substrates, such as lactate, or electron carriers, such as NADH (13). In S. oneidensis MR-1, there has been significant research on lactate dehydrogenases (14, 15), hydrogenases (16, 17), and formate dehydrogenases (18). However, to our knowledge, the NADH dehydrogenases have only been studied incidentally in whole-genome expression profiling, without specific gene deletion or biochemical studies. Four NADH dehydrogenases are encoded in the genome of S. oneidensis MR-1, with one predicted to pump protons (Nuo, SO_1009 to SO_1021), two predicted to pump sodium ions (Nqr1, SO_1103 to SO_1108; Nqr2, SO_0902 to SO_0907), and one predicted to be “uncoupling” and that does not translocate ions across the inner membrane (Ndh, SO_3517) (19). We note that the Nqr1 and Nqr2 labels are not used consistently across studies and genome databases, but we refer to them here as shown above.

Both sodium-pumping NADH dehydrogenases (Nqr1 and Nqr2) are found in all sequenced genomes in the Shewanella genus, while the proton-pumping NADH dehydrogenase (Nuo) has been found in only a few isolates, including S. oneidensis MR-1 (19). The same pattern has been observed with sodium- and proton-dependent flagellar stators, with S. oneidensis MR-1 being the only isolate known to contain the proton-dependent MotAB flagellar rotation system, while all sequenced Shewanella isolates contain the sodium-dependent PomAB system (20). Although this suggests that the sodium motive force (SMF) is the major energetic gradient used by S. oneidensis MR-1, other studies point to the importance of proton motive force (PMF). For example, the proton-specific uncoupler carbonyl cyanide *m*-chlorophenyl hydrazone (CCCP) induces biofilm dissolution in S. oneidensis MR-1 (21). Further, PMF generation by a light-driven proton pump improves current production and survival of S. oneidensis MR-1 in bioelectrochemical systems (22). Finally, we observed with BLAST (23) that the F_o_F_1_ ATP synthase of S. oneidensis MR-1 appears to be powered by PMF, due to the lack of specific residues necessary for sodium ion transport (24). Of course, PMF and SMF cannot be completely disentangled, because both are composed of two components, membrane potential (ΔΨ) and a concentration gradient of the coupling ion (ΔpH or Δ[Na^+^]). PMF and SMF are further connected by proton-sodium antiporters. The S. oneidensis MR-1 genome encodes several Na^+^/H^+^ antiporters, NhaA, NhaB, NhaC, NhaD, and two in the cation:proton antiporter-1 family (25). Therefore, it may be possible for S. oneidensis MR-1 to use sodium as the primary ion for respiratory coupling but convert SMF to PMF through antiporters for ATP synthesis. While this may appear to be an inefficient strategy, a recent review describes several organisms that utilize this method of oxidative phosphorylation, indicating that it may have advantages in some environments (26).

While NADH dehydrogenase activities have not been directly studied in S. oneidensis MR-1, some basic information about their function can be gleaned from the large amount of transcriptomic data collected for this organism. Several studies have detected significant changes in transcriptional regulation of the NADH dehydrogenases depending on the growth conditions. Rosenbaum et al. (27) found upregulation of the proton-pumping NADH dehydrogenase (*nuo*) in electrode-grown biofilms compared with aerobically grown planktonic cells. In contrast, Beliaev et al. (1) found decreased expression of *nuo* and *nqr2* (the operon containing *nqrA2* to *nqrF2*) with oxygen and metal oxide electron acceptors compared to fumarate. These studies show that NADH dehydrogenase expression is dependent on electron acceptor type, but they do not yet reveal a clear pattern.

Studies on known S. oneidensis MR-1 regulons also help predict when each NADH dehydrogenase is expressed. The regulation of Nqr NADH dehydrogenases appears to be based on carbon source type, energy levels within the cell, and the presence of specific electron acceptors. The *nqr2* operon is regulated by the hexose-dependent HexR regulator, suggesting that the expression of this NADH dehydrogenase depends on the available carbon source (28). In contrast, *nqr1* (the operon containing *nqrA1* to *nqrF1*) is not predicted to be part of the HexR regulon, but rather, it is regulated by the cAMP-dependent Crp regulator (29). The Fnr-like regulator EtrA also appears to play a role, leading to increased expression of *nqr2* under anoxic conditions and increased expression of *nqr1* under oxic conditions (30). Although it is unclear if Nqr1 and Nqr2 differ in function, due to their high sequence identity, differential regulation of each dehydrogenase suggests that they serve distinct roles in metabolism.

NADH is one of the most important electron carriers in cellular metabolism and appears to be critical for respiratory activity in S. oneidensis MR-1, based on the presence of genes encoding four different NADH dehydrogenases in its genome. However, electron transport from NADH to the quinol pool in S. oneidensis MR-1 has been understudied compared with terminal oxidases. To understand how S. oneidensis MR-1 couples growth to processes, such as electric current production and environmental metal cycling, it is essential to understand how PMF and SMF are generated during respiration. Therefore, we conducted a directed study on the role of each putative NADH dehydrogenase in the S. oneidensis MR-1 genome by generating in-frame deletions to disrupt each complex and by studying phenotypes of the mutant strains under oxic conditions. Our strategy was informed by a new analysis of an existing whole-genome fitness profiling data set.

## RESULTS

### Mining a whole-genome fitness data set for NADH dehydrogenase utilization patterns.

**FIG 1:**
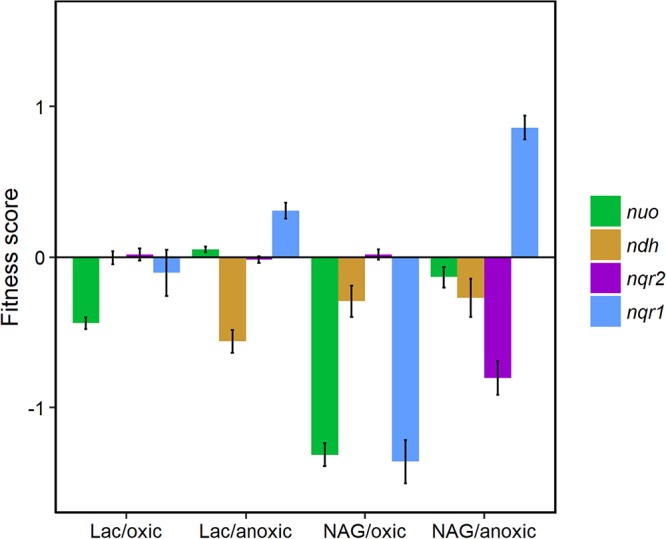
Fitness of NADH dehydrogenase mutants under several growth conditions: d,l-lactate/oxic (Lac/oxic), d,l-lactate/anoxic (Lac/anoxic), NAG/oxic, and NAG/anoxic. Values are averaged across loci in each operon and across multiple growth conditions that fit the descriptions mentioned above. Error bars represent the standard deviation. Fitness is relative to aerobic growth in LB. All data were generated by Deutschbauer et al. (31).

**FIG 2:**
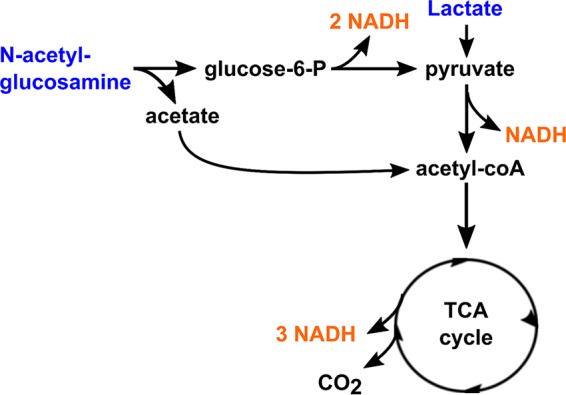
NAG and lactate metabolism in Shewanella oneidensis under aerobic conditions.

We analyzed an existing whole-genome fitness profiling data set from a study by Deutschbauer et al. (31) to gain initial insights into environmental conditions that influence the function of each NADH dehydrogenase encoded in the S. oneidensis MR-1 genome. In the study, a whole-genome transposon mutant library was grown under more than 200 different conditions. The fitness of the mutants was measured by changes in the abundance of sequence tags throughout the experiment. To focus on specific carbon sources, we plotted the fitness scores of NADH dehydrogenase mutant strains for a subset of 55 conditions where the library was grown in minimal medium under oxic or anoxic conditions with lactate or *N*-acetylglucosamine (NAG) as the substrate (Fig. 1). Conditions with differing sources of nitrogen, sulfur, and carbon (other than NAG and lactate) were excluded from the subset. Strains with mutations in the *nuo* (proton-dependent) and *nqr1* (sodium ion-dependent) genes showed the greatest fitness defects under oxic conditions. This effect was weaker with lactate than with NAG, which is unsurprising considering that lactate oxidation theoretically generates 4 NADH per molecule compared to 13 NADH per molecule for NAG (Fig. 2). Mutants with insertions in *ndh* (uncoupling) and *nqr2* (sodium ion-dependent) genes showed greater fitness defects under anoxic conditions than under oxic conditions. In general, all fitness defects were weak under anoxic conditions, which may be due to the lack of significant tricarboxylic acid (TCA) cycle activity in S. oneidensis MR-1 when oxygen is absent (32), resulting in less NADH generation from most substrates. Based on this analysis, we focused on oxic conditions, which showed the largest overall fitness defects for the NADH dehydrogenase mutants in the library. We chose two different carbon sources, sodium d,l-lactate and NAG, to understand the effects of substrates that generate differing amounts of NADH.

### Analysis of NADH dehydrogenase mutant growth and metabolism.

To study the impact of NADH dehydrogenase activity on growth and metabolism, we created an in-frame deletion in an essential gene in each putative NADH dehydrogenase-encoding operon in S. oneidensis MR-1, in *nuoN* (SO_1009), *ndh* (SO_3517), *nqrF1* (SO_1108), and *nqrF2* (SO_0907). Deletions were made in the terminal gene in each operon to restrict downstream pleiotropic effects while removing the function of each dehydrogenase. We cultured wild-type (WT) S. oneidensis MR-1 and the NADH dehydrogenase mutants in minimal medium in 24-well plates and measured growth via the optical density at 600 nm (OD_600_). Our measurements aligned well with observations from the whole-genome fitness analysis, confirming that culturing the mutants using d,l-lactate or NAG has differential effects on growth. The Δ*ndh* and Δ*nqrF2* mutants did not show growth defects with either NAG or d,l-lactate as a source of carbon under oxic conditions and therefore were omitted from further study (in Fig. S1 in the supplemental material).

**FIG 3:**
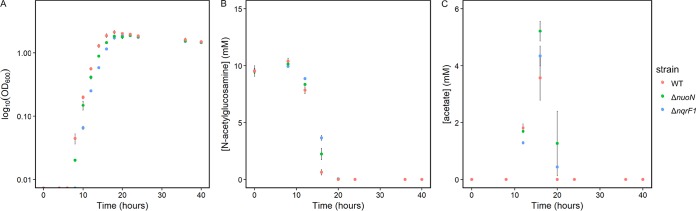
(A) Analysis of WT, Δ*nuoN* mutant, and Δ*nqrF1* mutant strains grown in 50 ml M5 minimal medium with 10 mM NAG in a 250-ml flask. (B and C) NAG (B) and acetate (C) concentration in culture supernatant.

Growth studies were repeated using a higher volume in flasks. As predicted by fitness data and growth in 24-well plates, both the Δ*nuoN* and Δ*nqrF1* mutants showed growth defects in minimal medium supplemented with 10 mM NAG as the carbon source (Fig. 3A). Both mutants had significantly decreased growth rates compared with the WT, as follows: 0.93 ± 0.05 s^−1^ for the WT, 0.76 ± 0.06 s^−1^ for the Δ*nuoN* mutant, and 0.81 ± 0.04 s^−1^ for the Δ*nqrF1* mutant (Table 1). To better understand the mechanism of the growth defect, the concentrations of substrates and metabolic by-products in the culture were monitored by high-performance liquid chromatography (HPLC) throughout growth. We observed that both mutants consumed significantly less NAG than WT at the 12-h (Δ*nqrF1* mutant, *P* ≤ 0.01; Δ*nuoN* mutant, *P* ≤ 0.05) and 16-h (Δ*nqrF1* mutant, *P* ≤ 0.001; Δ*nuoN* mutant, *P* ≤ 0.01) time points (Fig. 3B). Acetate accumulation in the cultures of both mutant strains was significantly higher than in cultures of WT at 20 h of growth (*P* ≤ 0.01), although all strains had consumed the excreted acetate by 40 h (Fig. 3C). No other major products were observed.

**TABLE 1:** Growth rate analysis of Shewanella oneidensis WT and NADH dehydrogenase mutants^*a*^

Carbon source	S. oneidensis strain	Growth rate (h^−1^)^*b*^	Difference from WT (%)	*P* value
NAG	WT	0.93 ± 0.05	NA	NA
	Δ*nuoN* mutant	0.76 ± 0.06	17.7	0.020
	Δ*nqrF1* mutant	0.81 ± 0.04	12.9	0.027
d,l-Lactate	WT	0.45 ± 0.05	NA	NA
	Δ*nuoN* mutant	0.41 ± 0.04	9.1	0.33
	Δ*nqrF1* mutant	0.43 ± 0.05	4.8	0.60
Acetate	WT	0.55 ± 0.04	NA	NA
	Δ*nuoN* mutant	0.36 ± 0.02	35.0	0.0018
	Δ*nqrF1* mutant	0.32 ± 0.01	42.8	0.0005

a Strains were grown in 50 ml of M5 minimal medium with 10 mM NAG or 20 mM d,l-lactate in 250-ml flasks and in 1 ml M5 with 10 mM acetate in a 24-well plate. NA, not applicable.

b Growth rates were calculated using the R package ‘growthcurver,’ which fits the growth curve data to the best-fit logistic curve (48).

**FIG 4:**
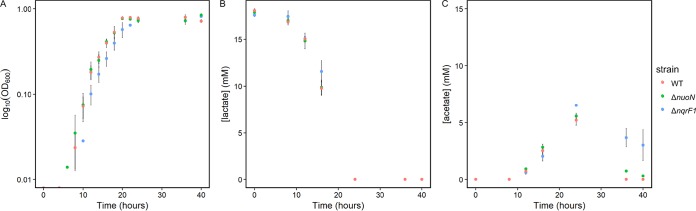
(A) Analysis of WT, Δ*nuoN* mutant, and Δ*nqrF1* mutant strains grown in 50 ml M5 minimal medium with 20 mM d,l-lactate in a 250-ml flask. (B and C) d,l-Lactate (B) and acetate (C) concentration in culture supernatant.

Flask growth experiments were also performed using 20 mM d,l-lactate as the substrate. Growth rates were not significantly different from the WT for either the Δ*nqrF1* or Δ*nuoN* mutant (Table 1). However, the Δ*nqrF1* mutant showed a distinct delay in growth, although all cultures were inoculated to the same cell density at the same time (i.e., the overall OD_600_ is significantly lower than WT from 12 to 16 h). This effect is less pronounced than the growth rate defect with NAG but was repeatable (Fig. 4A and S1). Similar to growth, d,l-lactate consumption and acetate accumulation by the Δ*nuoN* mutant were essentially indistinguishable from those of the WT; however, the Δ*nqrF1* mutant displayed subtle differences from the WT. The Δ*nqrF1* mutant used d,l-lactate more slowly than WT during early logarithmic growth (Fig. 4B). However, by 24 h, both the WT and the Δ*nqrF1* mutant consumed all available lactate (Fig. 4B). The Δ*nqrF1* mutant also accumulated 25% more acetate than WT at 24 h and had not fully consumed it by 40 h (Fig. 4C).

**FIG 5:**
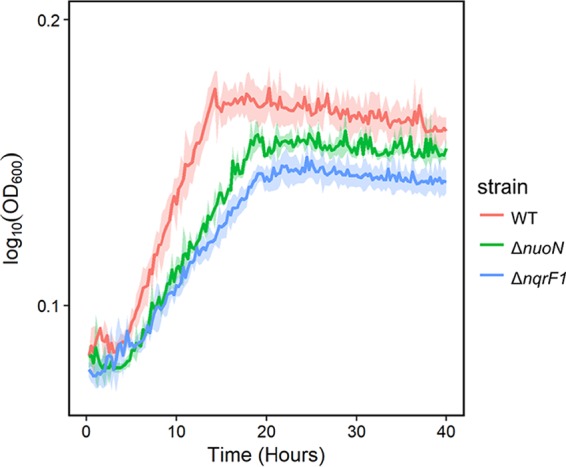
Growth of WT, Δ*nuoN* mutant, and Δ*nqrF1* mutant in 1 ml of M5 medium containing 10 mM sodium acetate in a 24-well plate.

Because we observed increased acetate accumulation by the mutant strains with both substrates, we hypothesized that they had a decreased capacity to consume acetate as a substrate compared to the WT. To determine if the mutant strains exhibited a reduced ability to utilize acetate, we conducted a 24-well growth experiment in minimal medium supplemented with 10 mM acetate (Fig. 5). Both the Δ*nuoN* and Δ*nqrF1* mutant strains exhibited growth defects compared to the WT under this growth condition (Table 1). The WT, Δ*nuoN* mutant, and Δ*nqrF1* mutant grew at rates of 0.55 ± 0.04 s^−1^, 0.36 ± 0.02 s^−1^ (*P* ≤ 0.01), and 0.32 ± 0.01 s^−1^ (*P* ≤ 0.001), respectively. The Δ*nuoN* mutant reached a final OD_600_ that was 4% (*P* = 0.17) less than that of the WT, and the Δ*nqrF1* mutant reached a final OD_600_ that was 12% (*P* ≤ 0.01) less than that of the WT, potentially reflecting a reduced efficiency of the overall electron transport chain.

### NADH dehydrogenase mutants exhibit reduced acid tolerance.

**FIG 6:**
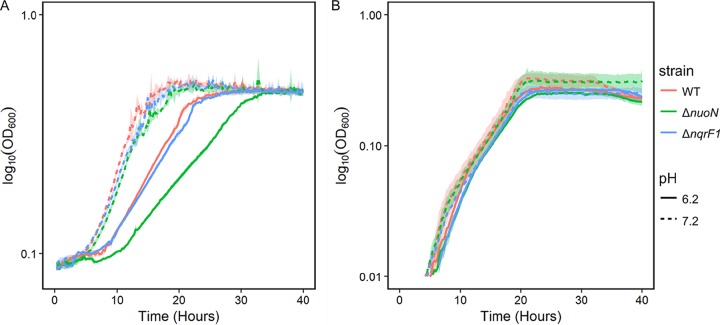
Growth of WT, Δ*nuoN* mutant, and Δ*nqrF1* mutant in 1 ml of M5 medium containing 10 mM NAG (A) or 20 mM d,l-lactate (B) at a pH of 6.2 or 7.2 in 24-well plates.

To exacerbate the effects of the deletions, we cultured the mutant strains in acidic medium to cause an additional burden on membrane potential. We compared growth in minimal medium at pH 7.2 or 6.2 with 10 mM NAG or 20 mM d,l-lactate as the carbon source (Fig. 6). Indeed, lower pH increased the defects of both mutant strains when grown with NAG. At the lower pH, the Δ*nuoN* mutant strain exhibited a stronger defect than the Δ*nqrF1* mutant strain. When grown with d,l-lactate, mutants grew similarly at pH 7.2 and pH 6.2, indicating that acid stress alone was not a strong enough stressor on membrane potential to cause changes in growth with a substrate producing minimal NADH and relying on quinone-linked dehydrogenases.

### A Δ*nuoN* Δ*nqrF1* double mutant is incapable of aerobic growth in minimal medium.

**FIG 7:**
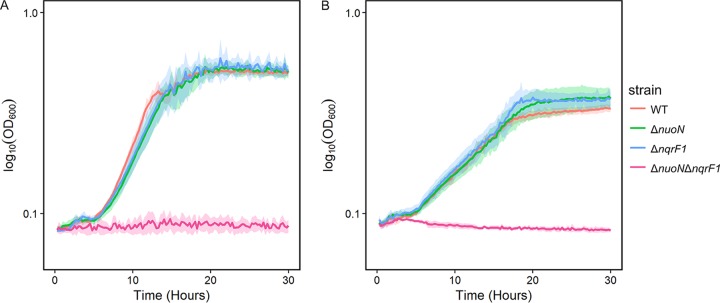
Growth of WT and Δ*nuoN*, Δ*nqrF1*, and Δ*nuoN* Δ*nqrF1* mutant strains in 1 ml of M5 medium containing 10 mM NAG (A) or 20 mM d,l-lactate (B) in 24-well plates.

Because both Δ*nuoN* and Δ*nqrF1* mutant strains exhibited only minor differences compared to the WT, we created a Δ*nuoN* Δ*nqrF1* double-knockout strain to determine whether these complexes have overlapping functions. In contrast to the single mutants, this double mutant is incapable of aerobic growth in in minimal medium supplemented with either 10 mM NAG or 20 mM d,l-lactate in 24-well plates (Fig. 7). We also attempted to grow this strain in the same medium in flasks and observed no change in OD_600_ over time (data not shown). Even in LB medium, this strain exhibited severely reduced growth compared with the WT (Fig. S2). Because the Δ*nuoN* Δ*nqrF1* mutant strain was unable to grow in minimal medium, we did not conduct additional analyses on its phenotype. We complemented the double-mutant strain with either *nuoN* or *nqrF1* expressed in *trans* from a multicopy plasmid. We observed enhanced growth rates in LB for both complemented strains compared with the Δ*nuoN* Δ*nqrF1* mutant carrying a plasmid with green fluorescent protein (GFP) (Fig. S3). This indicates that the growth defect observed for the double mutant was due to the absence of these genes, rather than being an off-target effect.

## DISCUSSION

### Implications of growth defects in NADH dehydrogenase mutant strains.

The growth deficiencies in the single and double knockout strains show that both Nuo and Nqr1 are important for aerobic growth and metabolism in S. oneidensis MR-1. The subtle phenotypes of the single-mutant strains indicate that the two complexes have significant functional overlap and that it is not necessary for both to function under the tested conditions. However, the severe growth defect of the double-mutant strain indicates that Nqr2 and Ndh are unable to compensate for the combined loss of Nuo and Nqr1, indicating that at least one is necessary. We hypothesize that Ndh cannot compensate because it does not generate PMF or SMF, and that Nqr2 cannot compensate because it is not significantly expressed under aerobic conditions. Our data suggest that Nqr1 plays a greater role than Nuo under the tested conditions due to the more significant phenotypes of the Δ*nqrF1* mutant strain. This is unsurprising, given the distribution of each of these in the genus, as discussed in the introduction. However, Nuo may play a more important role in managing acid stress, considering that at pH 6.2, a greater growth defect was observed for the Δ*nuoN* mutant than for the Δ*nqrF1* mutant. These data suggest that although the two NADH dehydrogenases are likely used concurrently during aerobic respiration, they also have distinct roles.

### Changes in metabolism suggest inhibition of the TCA cycle by increased intracellular NADH.

We observed that carbon sources that must be processed by the TCA cycle (and therefore, generate NADH) exacerbate the growth defect in the single-mutant strains. Metabolism of both NAG and acetate relies primarily on NADH as the electron carrier to feed the electron transport chain, while lactate may be partially oxidized by quinone-linked dehydrogenases that bypass NADH. We observed that growth with lactate was much less sensitive to the NADH dehydrogenase deletions than growth with the other substrates. It is somewhat surprising that growth defects were stronger with acetate than with NAG, although this may be explained by the possibility of generating formate through pyruvate formate lyase during NAG (or lactate) metabolism but not during acetate metabolism. Acetate metabolism may be further hindered by allosteric reduction of the activities of TCA cycle enzymes by high levels of NADH in the mutant strains. Although we have no direct measurements of intracellular NADH levels, accumulation of extracellular acetate during growth on NAG and d,l-lactate suggests that insufficient TCA cycle activity in the mutants forces acetate excretion. In the case of NAG, the acetate may be produced by either the initial removal of the acetyl group or by the production of acetyl-coenzyme A (acetyl-CoA) after glycolysis; either way, the acetate is being excreted rather than being processed.

### Roles of sodium ion versus proton energetics in S. oneidensis MR-1.

We observed that Nuo and Nqr1 have overlapping functions but did not elucidate how electron flux is partitioned between them when both are present. Analysis of the Shewanella genus provides some evidence that more flux would be directed to Nqr1, which would align well with our growth observations. While most Shewanella isolates originate from marine environments, S. oneidensis MR-1 was isolated from a freshwater lake. This habitat difference seems to be reflected in Shewanella energetics (33). Shewanella spp. generally utilize only sodium-ion based energetics, which can be advantageous in a marine environment, but S. oneidensis MR-1 has gained complexes for proton-based energetics through horizontal gene transfer. For example, all sequenced genomes in the Shewanella genus encode sodium ion-dependent Nqr NADH dehydrogenases, but S. oneidensis MR-1 is one of only a few strains that encode the proton-dependent NADH dehydrogenase Nuo (19, 34). Similarly, S. oneidensis MR-1 is the only strain in the genus with both SMF- and PMF-driven flagellar machinery, while the rest of the genus only has the SMF-driven system (20). Previous studies have not observed strong defects for knocking out PMF-dependent systems (35, 36), and our results reveal slightly stronger defects for the Nqr1 mutants than the Nuo mutants. This suggests that sodium remains an important coupling ion in S. oneidensis MR-1 despite its acquisition of PMF-dependent machinery. The potential benefits of maintaining both systems in the S. oneidensis MR-1 genome remain unclear.

The coutilization of proton- and sodium-dependent energetics in S. oneidensis MR-1 raises the question of whether SMF and PMF could be conserved separately for different cellular functions. While the ΔΨ component is shared between PMF and SMF, the ΔpH and Δ[Na^+^] components can vary and thereby influence the relative activity of PMF- versus SMF-dependent processes. S. oneidensis MR-1 preferentially utilizes its sodium-ion dependent stator, but its ATP synthase does not contain the necessary residues for sodium ion transport, indicating that it is proton dependent (23, 24). This arrangement could allow S. oneidensis MR-1 to favor either motility or ATP synthesis by upregulating Nqr1 or Nuo, respectively. Antiporters may also be utilized to interconvert between ΔpH and Δ[Na^+^] and thereby allow adaptive utilization of ion gradients for different functions (25, 37). Further study is needed to determine whether S. oneidensis MR-1 utilizes its coupling ion flexibility to conserve energy for different purposes.

### Implications of Na^+^-dependent energetics for bioelectrochemical technologies.

The ability to use a sodium-based respiratory system may have wide-ranging impacts on the physiology of S. oneidensis MR-1 in bioelectrochemical systems, particularly because local pH extremes near electrodes can be a major limiting factor (38). Previous analysis of biocathode processes has suggested that sodium ion-based respiration may be advantageous for organisms on a biocathode because of high local pH at the electrode (38). The localized alkaline pH at the biocathode can lead to a loss of PMF in the biofilm and hinder proton-dependent respiration (39). Therefore, S. oneidensis MR-1 may represent a promising chassis for engineering cathodic bioelectrochemical technologies, such as microbial electrosynthesis. Understanding the interactions between pH and the NADH dehydrogenases at the electrode would also allow us to better explore the genetic optimization of organisms in bioelectrochemical systems in general.

### Perspectives for future work.

To better understand the specific function of each dehydrogenase, it is necessary to generate triple-knockout strains, leaving only one functional NADH dehydrogenase in the genome, wherever possible. Membrane preparations from such strains could be used to confirm activity of each of these dehydrogenases through biochemical assays. If these strains are nonviable, a recently developed clustered regularly interspaced short palindromic repeat (CRISPR) interference system could be utilized to knock down the expression of the NADH dehydrogenases (40). This study focused on Nqr1 and Nuo mutants because the NqrF2 and Ndh mutants showed no growth defects under oxic conditions; however, we hypothesize that the NqrF2 and Ndh mutants will exhibit growth defects under anoxic conditions. Future study of all four mutant strains under anoxic conditions would also provide the opportunity to explore a wider range of thermodynamic constraints on metabolism, which may influence respiratory efficiency.

### Conclusion.

Under the laboratory conditions tested, S. oneidensis MR-1 utilized both Nqr1 and Nuo to oxidize NADH and conserve energy during aerobic growth. While single mutants lacking the activity of either complex grew only slightly slower than the WT, a double knockout was completely incapable of growth in minimal medium. Although two additional NADH dehydrogenases are encoded in the genome, either Nuo or Nqr1 was required for aerobic growth in minimal medium. Changes in the accumulation of acetate suggest that when either of these complexes is absent, intracellular NADH levels increase and potentially inhibit TCA cycle activity. We suggest that the coutilization of Nuo and Nqr results in adaptive metabolic redundancy and may represent a mechanism by which S. oneidensis MR-1 could conserve energy for different purposes, such as motility (preferentially sodium ion dependent) and ATP generation (likely proton dependent).

## MATERIALS AND METHODS

### Analysis of a whole-genome fitness profiling data set.

Fitness data for S. oneidensis MR-1 transposon mutants were downloaded as a supplemental information file for the whole-genome fitness profiling study by Deutschbauer et al. (31). Fitness values were averaged across each NADH dehydrogenase operon and across groups of conditions. Values for libraries grown in minimal medium were plotted.

### In-frame deletion of loci from the S. oneidensis MR-1 genome.

Deletions of the target genes were made using the pDS3.0 nonreplicative vector, as previously described, and confirmed by PCR (41). Fusion products were made via PCR, generating tagged complementary sequence fragments that were subsequently linked following the crossover PCR protocol. Six primers were designed and used for each strain, with the unique tag sequences added to the 5i and 3i primers (Table 2). Fusion products were inserted into the pDS3.0 vector with T4 ligase and transformed into chemically competent Escherichia coli WM3064 cells via heat shock. The plasmids were transferred to S. oneidensis MR-1 via a conjugation protocol similar to that of Webster et al. (11). Primary conjugants were screened for gentamicin resistance, and insertion into the genome was confirmed via two PCRs, with one reaction with the FO and 3o primers, and another with the 5o and RO primers. Primary integrants were grown for 8 h in LB without NaCl and then plated on LB without NaCl and with 10% sucrose. Individual colonies were screened via PCR using FO and RO flanking primers (Table 2) to identify deletion mutants. To acquire the double-mutant strain, it was necessary for the resolution step of the protocol to be extended from 18 h to 22 h. At 18 h, the cells still maintained antibiotic resistance, indicating that the plasmid had not yet resolved out of the genome of most cells.

**TABLE 2:** Primers used for generation of in-frame deletion mutants

Gene	Primer	Sequence
*nuoN*	FO	CTCTCAAATAGAGCACTC
	RO	ACCAGCATCTCCCACATG
	5O	GCTCAATATGATTGCGGGGCT
	5i	GGGATGAACACACCATGTCAGTGTTGCAGTAACGC
	3O	ATCTACTCGGGTAGCGAAGTG
	3i	TGACATGGTGTGTTCATCCCCGGAGAAAGTCATTGCAGGGC
*nqrF1*	FO	CTGGCCTTCTTCCTCGGTAT
	RO	GCCTTAGCTGCATCAATCTCGG
	5O	AGGGATTGCGGTAGTTGTAGTGT
	5i	CACAGAATCACGCTGTGATTCAGGTCCAATTCAAGAAGCCG
	3O	CGCTTATCAGGTCCAAAACCCCA
	3i	GAATCACAGCGTGATTCTGTGTGTGAATTAGCCCAAGGT
*ndh*	FO	TACATATCTTCAACATGTTGATTAATA
	RO	CTGAATGAAAATTACATAATTGAAGGG
	5O	ATTTCATCGGTTAGTGTAGTTTG
	5i	ACCCATGACCACTAAAATAGAAATAACAACCTCAACAAACTCAT
	3O	CTTAAAAACCGCCCTACC
	3i	TCTATTTTAGTGGTCATGGGTTCATAAAGAACAAAACAGAGAGCT
*nqrF2*	FO	CGCTGTCGTTCTTCCTCGGTATGTG
	RO	GTTGCGGCTCAGCTTTTGCG
	5O	GTGCTAATGACCTTAGCCGTGCC
	5i	GGGCTTGAGAGATCCCACTACTTCCATCCGATAACC
	3O	CAACTTGATGCGCTGGTAGAGGTC
	3i	GTAGTGGGATCTCTCAAGCCCGATTAGGTTGTAAGT

### Growth conditions.

All strains were precultured in LB medium (Miller; Accumedia) for 16 h. Precultures were washed with M5 medium and standardized to an OD_600_ of 1.0 prior to use. All growth experiments utilized the following M5 minimal medium recipe: 1.29 mM K_2_HPO_4_, 1.65 mM KH_2_PO_4_, 7.87 mM NaCl, 1.70 mM NH_4_SO_4_, 475 μM MgSO_4_·7H_2_O, 10 mM HEPES, 0.01% (wt/vol) Casamino Acids, 1× Wolfe's mineral solution (Al was not included), and 1× Wolfe's vitamin solution (riboflavin was not included), with the pH adjusted to 7.2 with 1 M NaOH. Either NAG or sodium d,l-lactate was added to a final concentration of 10 mM or 20 mM, respectively, unless noted otherwise. We conducted an additional experiment to ensure that differences in sodium concentrations between NAG and d,l-lactate did not cause significant changes in growth (Fig. S4). We did not observe any significant difference; therefore, extra sodium was not routinely added to the medium with NAG.

High-throughput growth experiments were performed in clear 24-well culture plates (catalog no. SIAL0526; Sigma) using 1 ml M5 medium, with four replicates per strain. Wells were inoculated with 10 μl of standardized preculture (washed in M5 and diluted to an OD_600_ of 1) and incubated in a Synergy HTX plate reader (BioTek Instruments, Winooski, VT) at 30°C with maximal shaking amplitude and minimal shaking speed. The OD_600_ was recorded by the instrument at 15-min intervals. High-throughput growth pH experiments were performed in 24-well plates, as described above, with the following modifications: M5 was prepared with the pH adjusted to 6.2 with 1 M HCl prior to autoclaving, and 24-well plates were prepared with triplicate for each strain under each condition, M5 pH 7.2 and M5 pH 6.2.

Flask growth experiments were performed in 250-ml Erlenmeyer flasks using 50 ml of M5 medium supplemented with either 20 mM d,l-lactate or 10 mM NAG. Flasks were inoculated with 50 μl of standardized preculture and incubated in a floor shaker (catalog no. 12500; New Brunswick Scientific) at 30°C and shaking at 275 rpm. Cultures were grown in triplicate for 40 h. Growth was monitored by removing 1 ml every 2 h starting at 4 h postinoculation and measuring the OD_600_. Samples were stored at −20°C prior to preparation for HPLC analysis.

### HPLC analysis.

HPLC analysis was performed on a Shimadzu 20A HPLC, using an Aminex HPX-87H column with a Micro-Guard cation H^+^ guard column (Bio-Rad, Hercules, CA) at 55°C. Samples were analyzed using a flow rate of 0.6 ml/min, in 5 mM sulfuric acid with a 30-min run time. Eluent was prepared by diluting a 50% HPLC-grade sulfuric acid solution (Fluka) in Milli-Q water and then degassing the solution at 37°C for 3 to 5 days before use. Compounds of interest were detected by refractive index (RID-20A). Samples were prepared by centrifuging 1-ml samples taken from flask growth for 10 min at 13,000 × *g* in a microcentrifuge (Minispin Plus; Eppendorf) to remove cells. The supernatant was removed and transferred to a 2.0-ml glass HPLC vial. Standards were prepared at concentrations of 1, 2, 5, 10, and 20 mM for d,l-lactate, NAG, and sodium acetate. Samples were maintained at 10°C by an autosampler throughout analysis.

### Complementation.

The double-mutant strain was complemented using an isopropyl-β-d-thiogalactopyranoside (IPTG)-inducible plasmid, pRL814 (a generous gift from Robert Landick, University of Wisconsin, Madison). pRL814 was isolated from E. coli using an E.Z.N.A plasmid DNA kit (Omega Bio-Tek). Prepared plasmid DNA was linearized using NdeI and HindIII (New England BioLabs). S. oneidensis MR-1 genomic DNA was isolated using the UltraClean microbial DNA isolation kit (Mo Bio, Carlsbad, CA). Primers used to amplify *nuoN* or *nqrF1* from S. oneidensis MR-1 genomic DNA were generated using the NEBuilder tool (New England BioLabs, Ipswich, MA). Linearized pRL814 and *nuoN* or *nqrF1* were assembled using the NEBuilder high-fidelity DNA assembly kit (New England BioLabs) using the standard protocol (42). Following assembly, E. coli WM3064 chemically competent cells were transformed with either pRL814_*nuoN* or pRL814_*nqrF1*. WM3064 strains were then used in conjugation with S. oneidensis MR-1 into the Δ*nuoN* Δ*nqrF1* mutant strain. In parallel, WM3064 strains bearing unmodified pRL814 (which expresses GFP) were used in conjugation with wild-type MR-1 and the Δ*nuoN* Δ*nqrF1* mutant. The control and complemented strains were grown in LB medium containing 100 μM IPTG and 50 μg/ml spectinomycin. Growth experiments were conducted in 24-well plates, with a 1-ml culture volume, in LB medium containing 100 μM IPTG and 50 μg/ml spectinomycin. Wells were inoculated with 10 μl of standardized preculture (washed in LB plus 100 μM IPTG and 50 μg/ml spectinomycin and diluted to an OD_600_ of 1) and incubated in a Synergy HTX plate reader (BioTek Instruments, Winooski, VT) at 30°C with maximal shaking amplitude and minimal shaking speed. The OD_600_ was recorded by the instrument at 15-min intervals.

### Data analysis.

Analysis of growth and HPLC data was performed using RStudio (43) using the following packages: ggplot2 (44), reshape2 (45), dplyr (46), and TTR (47). Analysis of growth rates from flask growth experiments were performed using R package ‘growthcurver’ using default values, with background correction set to “min” (48).

## Supplementary Material

Supplemental material

## References

[1] Beliaev AS, Klingeman DM, Klappenbach JA, Wu L, Romine MF, Tiedje JM, Nealson KH, Fredrickson JK, Zhou J 2005 Global transcriptome analysis of *Shewanella oneidensis* MR-1 exposed to different terminal electron acceptors. J Bacteriol 187:7138–7145. doi:10.1128/JB.187.20.7138-7145.2005. 16199584 PMC1251602

[2] Shi L, Squier TC, Zachara JM, Fredrickson JK 2007 Respiration of metal (hydr)oxides by *Shewanella* and *Geobacter*: a key role for multihaem *c*-type cytochromes. Mol Microbiol 65:12–20. doi:10.1111/j.1365-2958.2007.05783.x. 17581116 PMC1974784

[3] Kotloski NJ, Gralnick JA 2013 Flavin electron shuttles dominate extracellular electron transfer by Shewanella oneidensis. mBio 4:e00553-12. doi:10.1128/mBio.00553-12. 23322638 PMC3551548

[4] Marsili E, Baron DB, Shikhare ID, Coursolle D, Gralnick JA, Bond DR 2008 *Shewanella* secretes flavins that mediate extracellular electron transfer. Proc Natl Acad Sci U S A 105:3968–3973. doi:10.1073/pnas.0710525105. 18316736 PMC2268775

[5] von Canstein H, Ogawa J, Shimizu S, Lloyd JR 2008 Secretion of flavins by *Shewanella* species and their role in extracellular electron transfer. Appl Environ Microbiol 74:615–623. doi:10.1128/AEM.01387-07. 18065612 PMC2227709

[6] Biffinger JC, Pietron J, Ray R, Little B, Ringeisen BR 2007 A biofilm enhanced miniature microbial fuel cell using Shewanella oneidensis DSP10 and oxygen reduction cathodes. Biosens Bioelectron 22:1672–1679. doi:10.1016/j.bios.2006.07.027. 16939710

[7] Rosenbaum MA, Bar HY, Beg QK, Segrè D, Booth J, Cotta MA, Angenent LT 2011 *Shewanella oneidensis* in a lactate-fed pure-culture and a glucose-fed co-culture with *Lactococcus lactis* with an electrode as electron acceptor. Bioresour Technol 102:2623–2628. doi:10.1016/j.biortech.2010.10.033. 21036604

[8] Bouhenni RA, Vora GJ, Biffinger JC, Shirodkar S, Brockman K, Ray R, Wu P, Johnson BJ, Biddle EM, Marshall MJ, Fitzgerald LA, Little BJ, Fredrickson JK, Beliaev AS, Ringeisen BR, Saffarini DA 2010 The role of *Shewanella oneidensis* MR-1 outer surface structures in extracellular electron transfer. Electroanalysis 22:856–864. doi:10.1002/elan.200880006.

[9] Carmona-Martínez AA, Harnisch F, Fitzgerald LA, Biffinger JC, Ringeisen BR, Schröder U 2011 Cyclic voltammetric analysis of the electron transfer of Shewanella oneidensis MR-1 and nanofilament and cytochrome knock-out mutants. Bioelectrochemistry 81:74–80. doi:10.1016/j.bioelechem.2011.02.006. 21402501

[10] Golitsch F, Bücking C, Gescher J 2013 Proof of principle for an engineered microbial biosensor based on Shewanella oneidensis outer membrane protein complexes. Biosens Bioelectron 47:285–291. doi:10.1016/j.bios.2013.03.010. 23584391

[11] Webster DP, TerAvest MA, Doud DFR, Chakravorty A, Holmes EC, Radens CM, Sureka S, Gralnick JA, Angenent LT 2014 An arsenic-specific biosensor with genetically engineered *Shewanella oneidensis* in a bioelectrochemical system. Biosens Bioelectron 62:320–324. doi:10.1016/j.bios.2014.07.003. 25038536

[12] Flynn JM, Ross DE, Hunt KA, Bond DR, Gralnick JA 2010 Enabling unbalanced fermentations by using engineered electrode-interfaced bacteria. mBio 1:e00190-10. doi:10.1128/mBio.00190-10. 21060736 PMC2975363

[13] Anraku Y 1988 Bacterial electron transport chains. Annu Rev Biochem 57:101–132. doi:10.1146/annurev.bi.57.070188.000533. 3052268

[14] Pinchuk GE, Geydebrekht OV, Hill EA, Reed JL, Konopka AE, Beliaev AS, Fredrickson JK 2011 Pyruvate and lactate metabolism by *Shewanella oneidensis* MR-1 under fermentation, oxygen limitation, and fumarate respiration conditions. Appl Environ Microbiol 77:8234–8240. doi:10.1128/AEM.05382-11. 21965410 PMC3233039

[15] Brutinel ED, Gralnick JA 2012 Preferential utilization of d-lactate by *Shewanella oneidensis*. Appl Environ Microbiol 78:8474–8476. doi:10.1128/AEM.02183-12. 23001660 PMC3497373

[16] Meshulam-Simon G, Behrens S, Choo AD, Spormann AM 2007 Hydrogen metabolism in *Shewanella oneidensis* MR-1. Appl Environ Microbiol 73:1153–1165. doi:10.1128/AEM.01588-06. 17189435 PMC1828657

[17] Kreuzer HW, Hill EA, Moran JJ, Bartholomew RA, Yang H, Hegg EL 2014 Contributions of the [NiFe]- and [FeFe]-hydrogenase to H_2_ production in *Shewanella oneidensis* MR-1 as revealed by isotope ratio analysis of evolved H_2_. FEMS Microbiol Lett 352:18–24. doi:10.1111/1574-6968.12361. 24372594

[18] Kane AL, Brutinel ED, Joo H, Maysonet R, VanDrisse CM, Kotloski NJ, Gralnick JA 2016 Formate metabolism in *Shewanella oneidensis* generates proton motive force and prevents growth without an electron acceptor. J Bacteriol 198:1337–1346. doi:10.1128/JB.00927-15. 26883823 PMC4859590

[19] Pinchuk GE, Hill EA, Geydebrekht OV, De Ingeniis J, Zhang X, Osterman A, Scott JH, Reed SB, Romine MF, Konopka AE, Beliaev AS, Fredrickson JK, Reed JL 2010 Constraint-based model of *Shewanella oneidensis* MR-1 metabolism: a tool for data analysis and hypothesis generation. PLoS Comput Biol 6:e1000822. doi:10.1371/journal.pcbi.1000822. 20589080 PMC2891590

[20] Paulick A, Koerdt A, Lassak J, Huntley S, Wilms I, Narberhaus F, Thormann KM 2009 Two different stator systems drive a single polar flagellum in *Shewanella oneidensis* MR-1. Mol Microbiol 71:836–850. doi:10.1111/j.1365-2958.2008.06570.x. 19170881

[21] Saville RM, Rakshe S, Haagensen JAJ, Shukla S, Spormann AM 2011 Energy-dependent stability of *Shewanella oneidensis* MR-1 biofilms. J Bacteriol 193:3257–3264. doi:10.1128/JB.00251-11. 21572002 PMC3133257

[22] Johnson ET, Baron DB, Naranjo B, Bond DR, Schmidt-Dannert C, Gralnick JA 2010 Enhancement of survival and electricity production in an engineered bacterium by light-driven proton pumping. Appl Environ Microbiol 76:4123–4129. doi:10.1128/AEM.02425-09. 20453141 PMC2897463

[23] Madden T 2013 The BLAST sequence analysis tool. The NCBI handbook, 2nd ed National Center for Biotechnology Information, Bethesda, MD.

[24] Schulz S, Iglesias-Cans M, Krah A, Yildiz O, Leone V, Matthies D, Cook GM, Faraldo-Gómez JD, Meier T 2013 A new type of Na^+^-driven ATP synthase membrane rotor with a two-carboxylate ion-coupling motif. PLoS Biol 11:e1001596. doi:10.1371/journal.pbio.1001596. 23824040 PMC3692424

[25] Thomas PD, Campbell MJ, Kejariwal A, Mi H, Karlak B, Daverman R, Diemer K, Muruganujan A, Narechania A 2003 PANTHER: a library of protein families and subfamilies indexed by function. Genome Res 13:2129–2141. doi:10.1101/gr.772403. 12952881 PMC403709

[26] Mulkidjanian AY, Dibrov P, Galperin MY 2008 The past and present of sodium energetics: may the sodium-motive force be with you. Biochim Biophys Acta 1777:985–992. doi:10.1016/j.bbabio.2008.04.028. 18485887 PMC2695506

[27] Rosenbaum MA, Bar HY, Beg QK, Segrè D, Booth J, Cotta MA, Angenent LT 2012 Transcriptional analysis of *Shewanella oneidensis* MR-1 with an electrode compared to Fe(III)citrate or oxygen as terminal electron acceptor. PLoS One 7:e30827. doi:10.1371/journal.pone.0030827. 22319591 PMC3271074

[28] Leyn SA, Li X, Zheng Q, Novichkov PS, Reed S, Romine MF, Fredrickson JK, Yang C, Osterman AL, Rodionov DA 2011 Control of proteobacterial central carbon metabolism by the HexR transcriptional regulator: a case study in Shewanella oneidensis. J Biol Chem 286:35782–35794. doi:10.1074/jbc.M111.267963. 21849503 PMC3195618

[29] Novichkov PS, Kazakov AE, Ravcheev DA, Leyn SA, Kovaleva GY, Sutormin RA, Kazanov MD, Riehl W, Arkin AP, Dubchak I, Rodionov DA 2013 RegPrecise 3.0–a resource for genome-scale exploration of transcriptional regulation in bacteria. BMC Genomics 14:745. doi:10.1186/1471-2164-14-745. 24175918 PMC3840689

[30] Cruz-García C, Murray AE, Rodrigues JLM, Gralnick JA, McCue LA, Romine MF, Löffler FE, Tiedje JM 2011 Fnr (EtrA) acts as a fine-tuning regulator of anaerobic metabolism in *Shewanella oneidensis* MR-1. BMC Microbiol 11:64. doi:10.1186/1471-2180-11-64. 21450087 PMC3078092

[31] Deutschbauer A, Price MN, Wetmore KM, Shao W, Baumohl JK, Xu Z, Nguyen M, Tamse R, Davis RW, Arkin AP 2011 Evidence-based annotation of gene function in *Shewanella oneidensis* MR-1 using genome-wide fitness profiling across 121 conditions. PLoS Genet 7:e1002385. doi:10.1371/journal.pgen.1002385. 22125499 PMC3219624

[32] Brutinel ED, Gralnick JA 2012 Anomalies of the anaerobic tricarboxylic acid cycle in Shewanella oneidensis revealed by Tn-seq. Mol Microbiol 86:273–283. doi:10.1111/j.1365-2958.2012.08196.x. 22925268

[33] Myers CR, Nealson KH 1988 Bacterial manganese reduction and growth with manganese oxide as the sole electron acceptor. Science 240:1319–1321. doi:10.1126/science.240.4857.1319. 17815852

[34] Dehal PS, Joachimiak MP, Price MN, Bates JT, Baumohl JK, Chivian D, Friedland GD, Huang KH, Keller K, Novichkov PS, Dubchak IL, Alm EJ, Arkin AP 2009 MicrobesOnline: an integrated portal for comparative and functional genomics. Nucleic Acids Res 38:D396–D400. doi:10.1093/nar/gkp919. 19906701 PMC2808868

[35] Pinchuk GE, Hill EA, Geydebrekht OV, de Ingeniis J, Zhang X, Osterman A, Scott JH, Reed SB, Romine MF, Konopka AE, Beliaev AS, Fredrickson JK, Reed JL 2010 Constraint-based model of *Shewanella oneidensis* MR-1 metabolism: a tool for data analysis and hypothesis generation. PLoS Comput Biol 6:1–8. 10.1371/journal.pcbi.1000822 PMC2891590 20589080

[36] Paulick A, Delalez NJ, Brenzinger S, Steel BC, Berry RM, Armitage JP, Thormann KM 2015 Dual stator dynamics in the *Shewanella oneidensis* MR-1 flagellar motor. Mol Microbiol 96:993–1001. doi:10.1111/mmi.12984. 25727785

[37] Padan E, Venturi M, Gerchman Y, Dover N 2001 Na^+^/H^+^ antiporters. Biochim Biophys Acta 1505:144–157. doi:10.1016/S0005-2728(00)00284-X. 11248196

[38] Desloover J, Arends JBA, Hennebel T, Rabaey K 2012 Operational and technical considerations for microbial electrosynthesis. Biochem Soc Trans 40:1233–1238. doi:10.1042/BST20120111. 23176460

[39] Mulkidjanian AY, Dibrov P, Galperin MY 2011 The past and present of the sodium energetics: may the sodium motive force be with you. Biochim Biophys Acta 1777:985–992. doi:10.1016/j.bbabio.2008.04.028. PMC2695506 18485887

[40] Cao Y, Li X, Li F, Song H 2017 CRISPRi-sRNA: transcriptional-translational regulation of extracellular electron transfer in *Shewanella oneidensis*. ACS Synth Biol 15:1679–1690. doi:10.1021/acssynbio.6b00374. 28616968

[41] Erhardt H, Steimle S, Muders V, Pohl T, Walter J, Friedrich T 2012 Disruption of individual *nuo*-genes leads to the formation of partially assembled NADH:ubiquinone oxidoreductase (complex I) in *Escherichia coli*. Biochim Biophys Acta 1817:863–871. doi:10.1016/j.bbabio.2011.10.008. 22063474

[42] Gibson DG, Young L, Chuang R-Y, Venter JC, Hutchison CA III, Smith HO 2009 Enzymatic assembly of DNA molecules up to several hundred kilobases. Nat Methods 6:343. doi:10.1038/nmeth.1318. 19363495

[43] R Core Development Team. 2015 R: a language and environment for statistical computing. R Foundation for Statistical Computing, Vienna, Austria http://wwwR-project.org/.

[44] Wickham H 2009 ggplot2: elegant graphics for data analysis. Springer, New York, NY.

[45] Wickham H 2007 Reshaping data with the reshape package. J Stat Softw 21:1–20.

[46] Wickham H, Francois R 2015 dplyr: a grammar of data manipulation. R package version 042-3. https://cran.r-project.org/web/packages/dplyr/index.html.

[47] Ulrich J 2017 TTR: Technical Trading Rules. R package version 023-2. https://cran.r-project.org/web/packages/TTR/index.html.

[48] Sprouffske K, Wagner A 2016 Growthcurver: an R package for obtaining interpretable metrics from microbial growth curves. BMC Bioinformatics 17:172. doi:10.1186/s12859-016-1016-7. 27094401 PMC4837600

