# Supplementary material for: Shewanella oneidensis MR-1 Utilizes both Sodium- and Proton-Pumping NADH Dehydrogenases during Aerobic Growth
Source: Appl Environ Microbiol. 2018 May 31;84(12):e00415-18. doi: 10.1128/AEM.00415-18 (PMC5981069; doi:10.1128/AEM.00415-18)
Supplement: Supplemental material [file supp_84_12_e00415-18__index.html]

Supplemental material 

# Shewanella oneidensis MR-1 Utilizes both Sodium- and Proton-Pumping NADH Dehydrogenases during Aerobic Growth

## Supplemental material

- Supplemental file 1 -

  Growth of WT, Δ*ndh*, and Δ*nqrF2* strains in 1 ml minimal medium (Fig. S1); growth of WT, Δ*nuoN*, Δ*nqrF1*, and Δ*nuoN* Δ*nqrF1* strains in LB medium (Fig. S2); growth of WT/pRL814 empty vector, Δ*nuoN* Δ*nqrF1*/pRL814 empty vector, the Δ*nuoN* Δ*nqrF1* strain complemented with pRL814\_*nuoN*, and the Δ*nuoN* Δ*nqrF1* strain complemented with pRL814\_*nqrF1* in 1 ml LB medium (Fig. S3); growth of WT, Δ*nuoN*, and Δ*nqrF1* strains in 1 ml minimal media (Fig. S4).

  PDF, 1.0M
