# Supplementary material for: Shewanella oneidensis MR-1 Utilizes both Sodium- and Proton-Pumping NADH Dehydrogenases during Aerobic Growth
Source: Appl Environ Microbiol. 2018 May 31;84(12):e00415-18. doi: 10.1128/AEM.00415-18 (PMC5981069; doi:10.1128/AEM.00415-18)
Supplement: Supplemental material [file AEM.00415-18_zam012188555s1.pdf]

Supplementary materials for '*Shewanella oneidensis* MR-1 utilizes both sodium- and proton-pumping NADH dehydrogenases during aerobic growth'

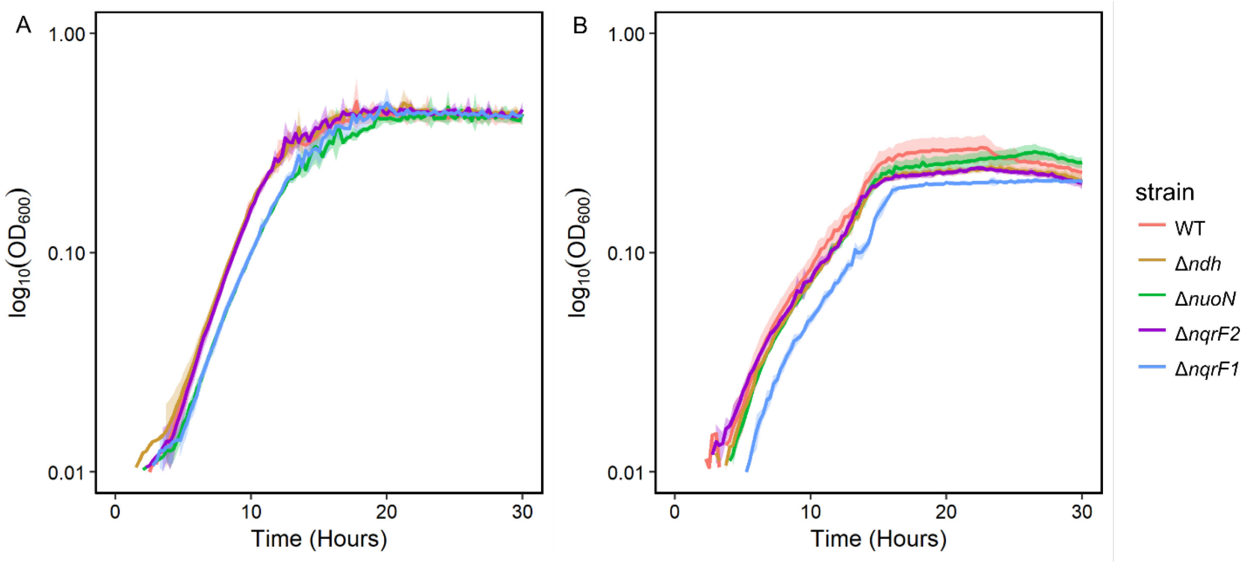

Figure S1. Growth of WT, Δ*ndh*, and Δ*nqrF2* in 1 mL minimal medium with (A) 20mM NAG and (B) 20 mM D,L-lactate. Growth was conducted in a 1 mL culture volume and monitored in 15 minute intervals in 24 well plates using the BioTek Synergy HTX plate reader (BioTek Instruments, Winooski, VT) at 30°C.

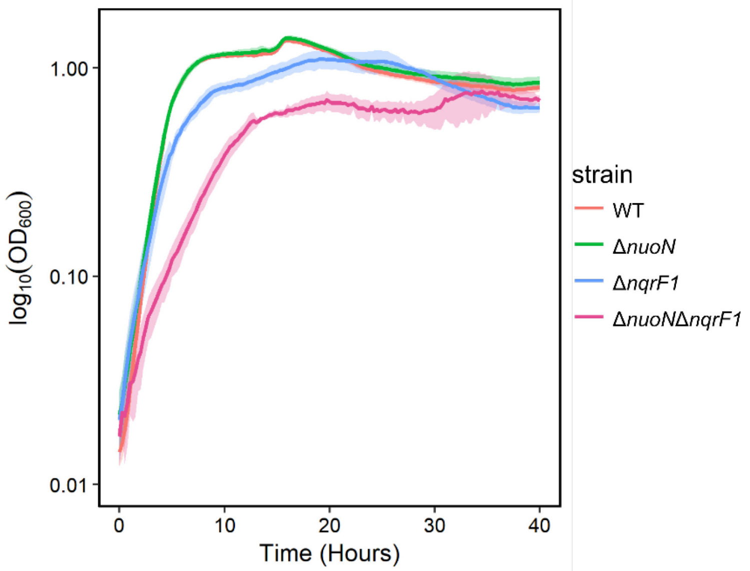

Figure S2. Growth of WT, Δ*nuoN*, Δ*nqrF1*, and Δ*nuoN*Δ*nqrF1* in LB medium. Growth was conducted in a 1 mL culture volume and monitored in 15 minute intervals in 24 well plates using the BioTek Synergy HTX plate reader (BioTek Instruments, Winooski, VT) at 30°C.

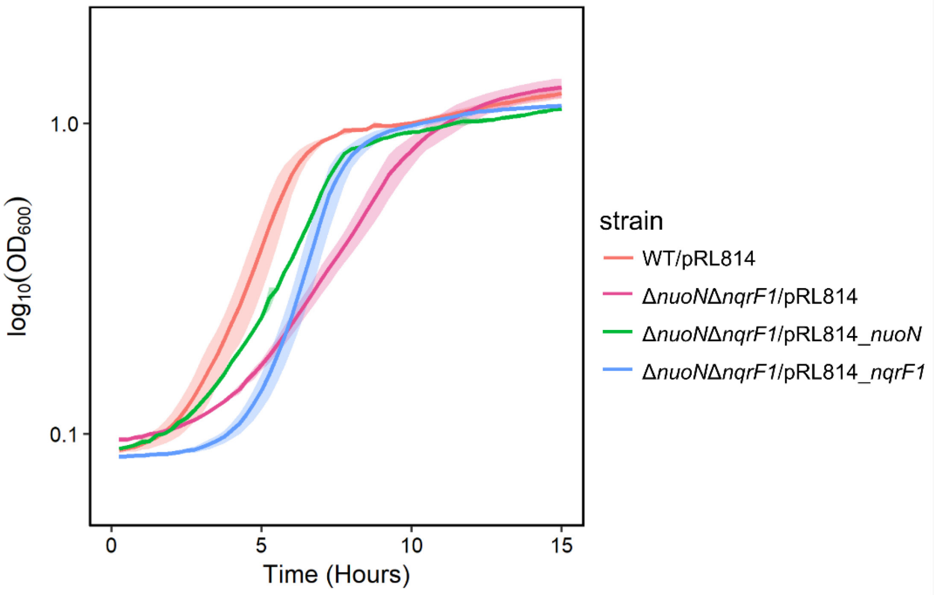

Figure S3. Growth of WT/pRL814 empty vector, Δ*nuoN*Δ*nqrF1*/pRL814 empty vector, Δ*nuoN*Δ*nqrF1* complemented with pRL814_*nuoN*, and Δ*nuoN*Δ*nqrF1* complemented with pRL814_*nqrF1* in 1 mL LB medium. Growth was conducted in a 1 mL culture volume and monitored in 15 minute intervals in 24 well plates using the BioTek Synergy HTX plate reader (BioTek Instruments, Winooski, VT) at 30°C.

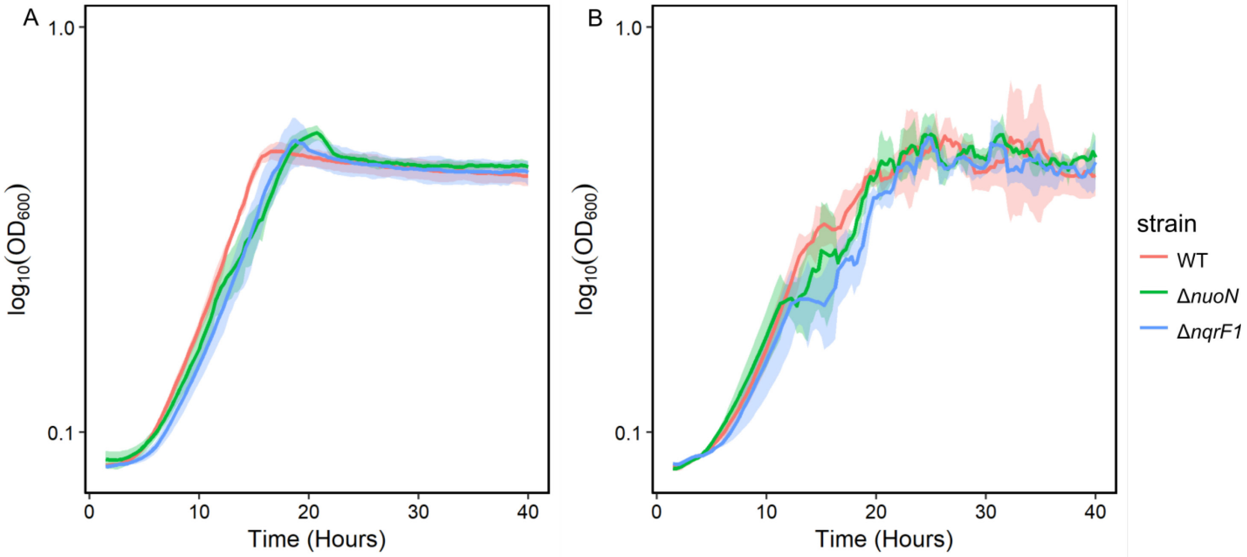

Figure S4. Growth of WT, Δ*nuoN*, and Δ*nqrF1* in 1 mL minimal media supplemented with (A) 10 mM NAG and (B) 10 mM NAG with additional 20 mM NaCl to normalize sodium for 24-well assays conducted with 20 mM sodium D,L-lactate. Growth was conducted in a 1 mL culture volume and monitored in 15 minute intervals in 24 well plates using the BioTek Synergy HTX plate reader (BioTek Instruments, Winooski, VT) at 30°C.
